# Complete Genome Sequence of the Poly-γ-Glutamate-Synthesizing Bacterium Bacillus subtilis Bs-115

**DOI:** 10.1128/genomeA.00197-18

**Published:** 2018-04-19

**Authors:** Fengqing Wang, Lijuan Gong, Lihong Zhou, Jinzhong Liang

**Affiliations:** a Department of Bioscience Engineering, Sichuan University of Science & Engineering, Zigong, People’s Republic of China; b Key Laboratory of Food Science and Engineering, College of Food Engineering, Harbin University of Commerce, Harbin, People’s Republic of China

## Abstract

Bacillus subtilis Bs-115 was isolated from the soil of a corn field in Yutai County, Jinan City, Shandong Province, People’s Republic of China, and is characterized by the efficient synthesis of poly-γ-glutamate (γ-PGA), with corn saccharification liquid as the sole energy and carbon source during the process of γ-PGA formation. Here, we report the complete genome sequence of Bacillus subtilis Bs-115 and the genes associated with poly-γ-glutamate synthesis.

## GENOME ANNOUNCEMENT

Bacillus subtilis Bs-115 was isolated from the soil of a corn field in Yutai County, Jinan City, Shandong Province, People’s Republic of China. Strain Bs-115 can use glucose and other common carbon sources for growth. Currently, there are many strains of this genus that can be retrieved in NCBI that have been reported to have poly-γ-glutamate (γ-PGA) synthesis ability. Strain Bs-115 was the only one that could use corn saccharification liquid as the only carbon and energy source for γ-PGA synthesis. γ-PGA is an extremely important substance with unique properties that have been exploited for a wide array of useful applications, such as in food, cosmetics, medicine, agriculture, oil recovery, and water treatment (1). The number of potential applications for γ-PGA is still increasing.

The genome sequence of Bacillus subtilis Bs-115 was determined by Nextomics Biosciences Co., Ltd. (Wuhan, People’s Republic of China), using PacBio single-molecule real-time sequencing technology. A total of 1,687,494,897 bp of original data was obtained. The depth of sequencing was about 367.31×, and the average length was 15,134 bp. Celera assembler and minumus2 software were used to assemble reads and connect contigs (2), respectively. The whole information map of the Bacillus subtilis Bs-115 genome was drawn using Circos (3) to obtain gene structure prediction results.

The genome of Bacillus subtilis Bs-115 consists of a single circular chromosome of 4,142,593 bp and has an average GC content of 43.35%. There are a total of 4,661 putative open reading frames (with an average size of 792.91 bp) according to Glimmer (4). Eighty-eight tRNA genes for all 20 amino acids and 10 16S-23S-5S rRNA operons were identified by tRNAscan-SE (5) and RNAmmer (6), respectively.

Sixteen simple sequence repeats (SSR) and 86 tandem repeats were identified by misa and TRF (7), respectively. DNA base modification (such as m4C and m6A type base methylation modification) plays an important role in biological processes, including growth and aging, immunity, pathogenicity of bacteria, and the development of disease. Surface modification analysis (8) showed that there were 388,984 m4C and 11,504 m6C base modifications. The consistencies of the restriction modification system of the genome with putative type I specificity, a putative type I methyltransferase system, a putative type I restriction enzyme, and a putative type IV methyl-directed restriction enzyme were 100%, 94.75%, 95.04%, and 99.23%, respectively.

The genome of Bacillus subtilis Bs-115 contains the *pgsBCAE* gene, which is related to γ-PGA synthase enzymes, and the *pgdS* and *ggt* genes, which are related to γ-PGA degradation enzymes. Much work has been done to improve γ-PGA production based on the heterologous expression of synthase genes. However, little research has been performed to investigate ways to improve the yield and molecular weight of γ-PGA by reducing the activity of degradation enzyme gene expression. In particular, research on *in situ* editing of the target gene (the γ-PGA degradation enzyme gene) to improve the production and molecular weight of γ-PGA using clustered regularly interspaced short palindromic repeat (CRISPR)-Cas genome editing technology has not yet been reported.

### Accession number(s).

The nucleotide sequence comprising the Bacillus subtilis Bs-115 genome was deposited in GenBank with the accession number CP020722.
